# The psychometric properties and temporal dynamics of subjective stress, retrospectively assessed by different informants and questionnaires, and hair cortisol concentrations

**DOI:** 10.1038/s41598-018-37526-2

**Published:** 2019-01-31

**Authors:** Lisa J. Weckesser, Friedericke Dietz, Kornelius Schmidt, Juliane Grass, Clemens Kirschbaum, Robert Miller

**Affiliations:** 1 Faculty of Psychology, Technische Universität Dresden, Dresden, Germany; 2 Unit Epidemiology, Statistics, and Exposure Modeling, Federal Institute for Risk Assessment, Berlin, Germany

## Abstract

To date, there is only scarce evidence for a considerable association of subjective and objective stress measures, which might be attributable to method bias (e.g., confounding) and/or asynchrony of their temporal changes. To validate different subjective stress measures by a physiological measure of long-term stress (hair cortisol concentrations; HCC), 37 heterosexual couples (*N* = 74) completed a 12-week internet-based assessment protocol comprised of a weekly hassle scale (WHS; once per week), a perceived stress scale (PSS; once per month), and a chronic stress scale (TICS; once after three months). Partners provided vicarious stress ratings. When averaged across time, self-reported WHS significantly predicted HCC (*r* = 0.27), whereas the PSS and TICS did not (*r* < 0.22). Dynamic factor analysis (i.e., state-space modelling) confirmed that WHS was the most valid indicator of subjective stress, explaining up to 16% of the variance in HCC (*r* = 0.37) with a time lag of ~4 weeks. This temporally delayed effect of subjective stress is consistent with the presumed retrospective character of HCC, but also suggests that the majority of variance in hair cortisol is attributable to other causes than subjective stress such as individual disposition to display increased adrenocortical activity.

## Introduction

**Figure 1:**
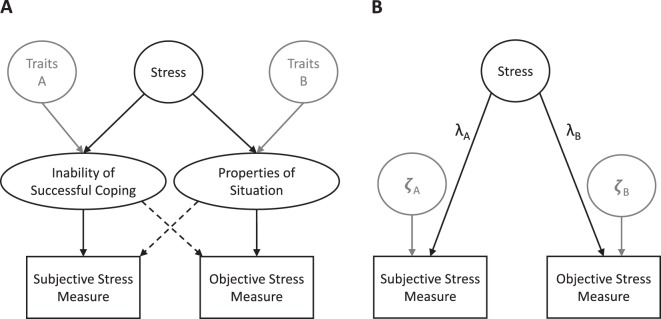
Path diagrams of (**A**) the causal model implied by the given definition of *stress*, and (**B**) a corresponding reflective measurement model. The two components of stress, that is, the (1) anticipated or perceived inability of successful coping and (2) the novelty, unpredictability, or uncontrollability of a situation are differentially affected by various stress-unrelated traits. Thus, stress should ideally be assessed based on the covariation between indicators of both components. The residual variances in these indicators (ζ) will accordingly comprise unsystematic measurement error and the systematic variance of the stress-unrelated confounding traits.

In their psychophysiological stress concept, Koolhaas and colleagues^1^ define *stress* as anticipated or perceived inability to successfully cope with unknown, unpredictable, and/or uncontrollable situations^2,3^. Since these situations specifically increase the secretion of the stress hormone cortisol, *stress* is inherently characterized by the co-occurrence of (1) a subjective appraisal component and (2) objectively quantifiable cortisol changes^4–6^. Thus, multimethod approaches that are comprised of both, subjective and objective stress measures arguably form the most robust strategy to assess *stress* and investigate its psychobiological sequelae. The causal model implied by the given definition of stress, and a corresponding measurement model are illustrated in Fig. 1.

Notably, each of the two stress components does not exclusively indicate *stress*, but concurrently reflects residual trait influences to varying extents. In this regard, the respective advantages and limitations of subjective and objective stress measures are briefly summarized below, highlighting why these measures do not necessarily predict stress-sensitive developmental and psychological health outcomes^2^. Based on the identified limitations, the present article strives to validate different stress questionnaires by an objective a physiological measure of long-term stress. Thus, recommendations are derived on how to best assess *stress* when physiological markers cannot be obtained due to infrastructural or financial restrictions.

## Subjective Stress Measures

The subjective appraisal component of *stress* is usually measured by self-report measures such as the Perceived Stress Scale (PSS)^7^, or the Trier Inventory of Chronic Stress (TICS)^8^. Notably, the scores of these questionnaires have demonstrated very good reliability^9^ as compared to the more direct and traditional approach of subjective *stress* assessment using hassles scales^10,11^. Nonetheless, questionnaire data are inherently prone to different kinds of method bias which may compromise their predictive value (i.e., the validity of their interpretations) despite of good reliability^12,13^.

First, confounding psychological traits like low self-efficacy or high neuroticism have been shown to bias stress assessments that rely on self-report measures (see Fig. 1A)^14,15^. The remarkably large associations between these confounding traits and stress measures (*r*’s > *0.5*) have often been interpreted as supportive of the convergent validity of different subjective stress measures. However, these findings simultaneously question the discriminant validity of subjective stress assessment because the majority of variance is not actually attributable to stressful properties of the situation. To alleviate such a confounding, the assessment of subjective stress may benefit from the consideration of different informant perspectives^16^, but information about the practical impact of such vicarious ratings with regard to stress questionnaires is lacking so far.

Second, most stress questionnaires rely on the retrospective report of perceived stress across comparably long time intervals (i.e., up to several months). An obvious drawback of retrospective reports relates to their probable sensitivity to recall bias which increases with longer time intervals^17,18^. Recall bias can be alleviated by the assessment of subjective stress during shorter time intervals (e.g., using hassles scales). Particularly in changing environments, shorter time intervals are supposed to provide more valid, but simultaneously more occasion-dependent information about subjective stress as compared to questionnaires like the PSS or the TICS. In order to match the higher stability of retrospective reports across long time intervals, data from the shorter time intervals must therefore be sampled more often and aggregated across time^19^. Due to technological progress, computer-assisted assessment in ambulatory settings has recently become a popular and readily implementable strategy to realize such high-frequency sampling schedules^20^. Similar to the missing data on different informant perspectives, however, researchers and practitioners are still lacking information about the presumed gain in validity when they rely on such stress assessment protocols.

## Objective Stress Measures

The objective component of *stress* is commonly measured by the more expensive, biochemical quantification of cortisol in different biological specimens^1^. These measures are characterized by a considerably higher reliability (i.e., *R*^2^ ~ 97%)^21,22^ and lower potential for measurement bias^23^ than the above-mentioned subjective stress measures. During the last decades, saliva was arguably the most famous type of specimen to determine cortisol concentrations^24^. However salivary cortisol is comprised of 42% to 88% variance due to within-day influences^21,25^, which highlights its very pronounced sensitivity to short-term environmental changes. This large variability tremendously constrains the validity of salivary cortisol as an objective stress measure. For example, the sum score of the 10-item version of the Perceived Stress Scale (PSS) was previously reported to yield a minimal retest stability of r_tt,1_ = 0.72 (see *section 2.3*). By contrast, the minimal retest stability for morning salivary cortisol amounts to r_tt,2_ = 1–88% = 0.12. Thus, the lower bound of the maximally observable correlation between the PSS and morning salivary cortisol is r_max_ = (r_tt,1_ × r_tt,2_)^0.5^ = 0.29^12,26^. Such low validity bounds can only be increased when salivary cortisol is sampled frequently (i.e., several times a day) and aggregated across time^27^.

In order to bypass such costly assessment schedules, hair cortisol (HCC) was recently recommended as a more suitable measure of objective stress^28,29^. This recommendation is based on the assumption that HCC predominately reflects the accumulated cortisol secretion over longer time periods, which is reflected by lower variance portions of ~8% and 15% to 39% attributable to within-day and within-year influences, respectively^22,30^. Moreover, a considerable portion of variance is shared between HCC and salivary cortisol (R^2^ = 37%) when the latter is aggregated across 30 days^31^. Irrespective of these excellent assessment properties, however, objective stress measures based on cortisol cannot be interpreted in isolation because they are also sensitive to stress-unrelated psychophysiological traits (see Fig. 1A)^32,33^.

## Multimethod Stress Assessment

Proceeding for the given definition of stress, the utility (and cost efficiency) of multimethod assessments of *stress* implicitly relies on the assumption that subjective and objective stress measures both reflect a *common cause* of stress-related psychophysiological changes within an individual (as shown in Fig. 1)^29,34^. If this common cause hypothesis is true, the aggregation of subjective and objective stress measures will yield a combined stress measure that is characterised by an increased ratio of true stress-related variance relative to residual (error and method) variance. Thus, the validity of the multimethod stress assessment will increase.

Support for the common cause hypothesis was primarily derived from studies contrasting stressed and non-stressed individuals. For instance, caregivers and unemployed robustly display elevated subjective stress and higher (saliva and hair) cortisol concentrations as compared to control individuals^29,35–37^. Apart from these findings, however, the overall association between subjective (i.e., self-reported) stress and objectively quantifiable cortisol concentrations at the same assessment occasion rarely exceeds correlation coefficients of *r* = 0.20^29,34,38^. While the above outlined properties and limitations of the different subjective and objective stress measures may in part explain this considerable lack of convergent validity, another reason was highlighted by Schlotz and colleagues who found that the stress–related changes in mood and plasma/salivary cortisol were not perfectly synchronized in time^34^. More specifically, the association of mood and salivary cortisol only reached its peak of *r* = 0.54 when a temporal offset of ~12 min in between these measures was accounted for^33^. Because HCC can only reflect the stress levels during the hair growth period (on average 1 month ~1 cm)^39^, its convergent validity probably also depends on the time period that is covered by the respective subjective stress measure. To the best of our knowledge, however, such data are also lacking to date.

## Research Objectives

Proceeding from these considerations, the present study was designed to investigate (1) the extent to which subjective stress is reflected by different questionnaires (covering varying time intervals). Accordingly, we strived to identify the questionnaire that was the purest indicator of their joint, stress-related covariance. As the validity of stress assessments based on self-report questionnaires may be compromised by confounding traits, the study also investigated (2) if the covariance between stress questionnaires differed as a function of informants. To further determine if this stress-related covariance was actually predictive of long-term cortisol secretion, (3) the temporal dynamics between changes in subjective stress and the objective stress measure HCC were assessed across different assessment occasions. To this end, a total sample of 74 individuals (i.e., 37 heterosexual couples) completed a 12-week internet-based assessment procedure: Subjective stress was assessed using a weekly hassles scale (WHS; once per week), the PSS (once per month)^40^, and the screening scale of chronic stress from the TICS (once after three months)^8^. Finally, all participants provided a hair sample that was garnered by trained medical personnel in our laboratory. Based on a presumed hair growth rate of one centimetre per month, HCC was measured in the three most proximal centimetres of scalp hair. Thus, HCC was supposed to reflect the (cumulative) cortisol exposure during the last three months (i.e., the time interval that was covered by the whole assessment procedure).

## Material and Methods

### Participants

**Figure 2:**
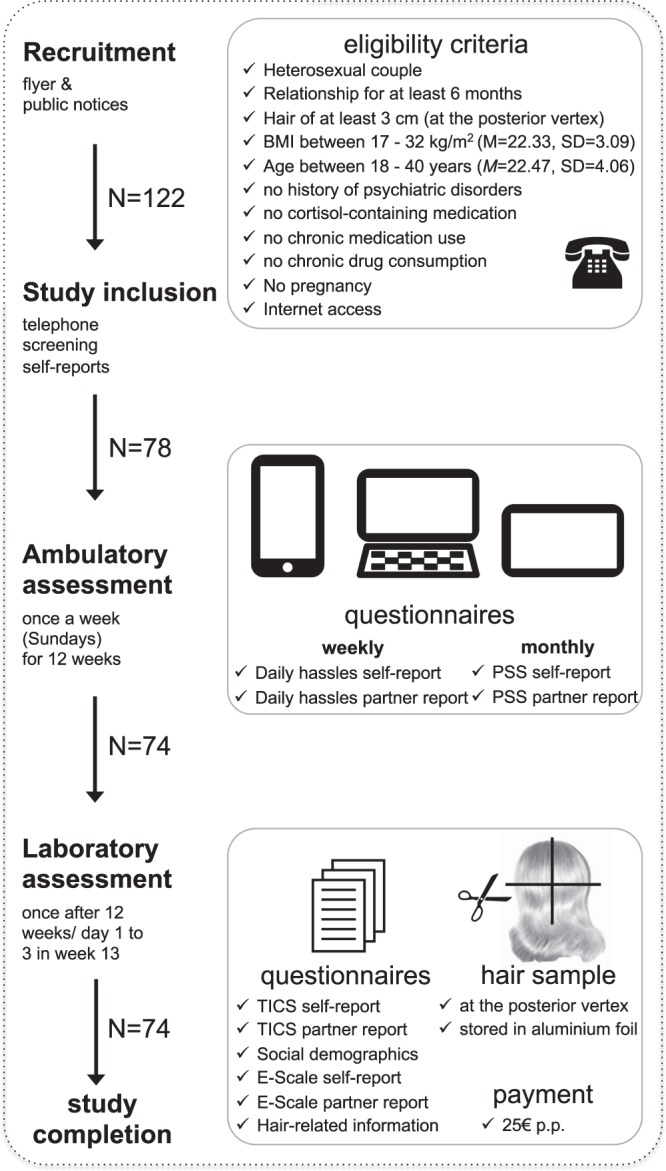
Study design and procedure.

As shown in Fig. 2, we initially recruited 122 young men and women (i.e., 61 couples) using public notices and flyers in the Dresden area. 78 of these participants (63.93%) fulfilled all eligibility criteria (see Fig. 2) and were admitted after providing their written informed consent. The final sample was comprised of *N* = 74 of these participants, because of one separation and one pregnancy that were reported in the course of the study. All participants were of Caucasian descent. Baseline characteristics of the sample are reported in Table 1. Each couple received a total compensation of 50 € for participation. The study was conducted in accordance with the Declaration of Helsinki and approved by the ethics committee of the TU Dresden (IRB00001473/IORG0001076).

**Table 1:** Baseline characteristics of the final sample (N = 74).

	Q_25_	Median (Q_50_)	Q_75_	%
age (yrs)	20	21	24	—
portion of females	—	—	—	50.0
hormonal contraception (females)	—	—	—	75.7
height (cm)	168	175	181	—
weight (kg)	59	67	75	—
blonde hair color	—	—	—	46.0
weekly sport activity	—	—	—	36.5
time spent outside (hrs/d)	1.5	2.5	3.4	—
daily alcohol consumption	—	—	—	8.1
daily smoking	—	—	—	4.1
net income (€/month)	775	1200	1825	—
academic education				
none	—	—	—	17.6
undergraduate	—	—	—	56.8
Bachelor’s degree	—	—	—	14.9
Master’s degree	—	—	—	10.8

### Study Design And Procedure

This study was implemented using a within-participant, repeated measurement design. After admittance, each participant completed an internet-based 12-week assessment protocol (realised with SoSci Survey®) using his/her own smartphone, tablet, or personal computer^41^. Every Sunday evening at 18.00 o’clock, each participant received an email with a personalized link to his/her questionnaires (i.e., PSS and/or WHS). If the questionnaires were not completed within 3 hours, they received an automated reminder on Sunday at 21.00 o’clock, and if required, again on Monday at 18.00 o’clock. Within the first three days of week 13 (i.e., immediately after the assessment period was completed), all participants were invited to the biopsychological laboratory of the *Technische Universität Dresden* to donate a hair sample, fill out two additional questionnaires (i.e., the TICS and an empathy scale; see *section 2.3*), and finally provide further hair-related information.

### Questionnaires

To obtain information from multiple informants, all participants provided each of the subjective stress measures reported below twice at each assessment occasion; once for themselves (self-report) and once for their partners (partner-report)^13,42^. Formerly published psychometric properties of these measures are presented in this section, whereas their corresponding estimates in the present study are reported in section 3.1.

#### Weekly Hassle Scale

Once per week, participants completed a 30-item *Weekly Hassle Scale* (WHS). The 30 items were compiled from the *Revised Daily Hassle Scale*^43^, the *Revised University Student Daily Hassle Scale*^44^, and a list of everyday stressors (*Liste Alltäglicher Streßereignisse*; please see Appendix A for further information)^45^. Using a 5-point Likert scale, participants first indicated how often each particular hassle occurred during the last week (from 0 = *did not occur* to 4 = *occurred always*) and if it occurred, how stressful it has been for them or their partners, respectively (from 0 = *not at all stressful* to 4 = *extremely stressful*). Three almost collinear (*r*’s > 0.86) stress measures were calculated from these data: The number of hassles that occurred at least once during the preceding week (WHS_n_), the mean score of all occurred weekly hassles (WHS_occur_), and the mean score of the perceived stressfulness of these weekly hassles (WHS_intens_). WHS_occur_ was selected as primary measure because it provided more fine-graded information than WHS_n_ while being putatively less susceptible to confounding as compared to WHS_intens_.

#### Perceived Stress Scale

Once per month, participants filled out the German 10-item version of the Perceived Stress Scale (PSS) provided at http://www.psy.cmu.edu/∼scohen/scales.html. The PSS asks how often a person has felt stressed, surprised, unable to cope, or out of control in the last month (from 0 = *never* to 4 = *very often*) and is supposed to provide a general measure of perceived stress^7^. The PSS has been previously reported to exhibit a retest stability of 0.72 < *r*_*tt*_ < 0.86 (up to one month) and an internal consistency of 0.74 < *α* < 0.91^9^.

#### Trier Inventory of Chronic Stress

Once after three months, all participants completed the screening scale of chronic stress (12 Likert-scaled items ranging from 0 = *never* to 4 = *very often*) from the Trier Inventory of Chronic Stress (TICS)^8^. The remaining TICS subscales are not investigated here due to their lacking associations with HCC in previous studies^38^. The TICS chronic stress subscale was reported to display an internal consistency of *α = *0.91^8^. To the best of our knowledge, there are no data about any retest stability of the TICS available so far^46^.

#### Empathy Scale

To exploratory check if the coherence of the self- and the partner-reports depended on the individual disposition to experience stress vicariously^47^, a German empathy scale (ES) was implemented as well (cf. Fig. 2)^48^. The ES was comprised of 11 items on *empathy* (e.g., “*I can easily emulate the feelings of a fictional character*”) and 12 items on *concerns* about others and their presumed emotions (e.g., “*It makes me sad to see a single person in a group of people*”). Responses are given on a Likert scale (from 0 = *not true* to 4 = *true*) and aggregated to a sum score. The ES is characterised by a retest stability of *r*_*tt*_ = 0.90 and an internal consistency of *α* = 0.92^48^. For reasons of clarity and due to the negligible impact of the ES on the results of this study, the corresponding data are only provided as supplementary material.

### Hair Cortisol Analysis

To obtain an objective stress measure, HCC was quantified in the three most proximal centimetres of the gathered scalp hair, which was prepared and analysed as proposed by Gao and colleagues^49^. In short, each hair strand was repeatedly washed using isopropanol, dried for 6 hours, and thereafter analysed in triplicates based on three segments of 10 mg of whole, non-pulverized hair. Cortisol was extracted by incubating each specimen for 18 hours in 1800 μL methanol. 1600 μL of the resulting suspensions were purged with nitrogen at 50 °C and a pressure of 0.1 bar for at least 40 min. Thereafter, the supernatants were resuspended in 225 μL of distilled water and submitted to liquid-chromatography coupled to tandem-mass spectrometry (LC-MS/MS). All mass chromatograms are available from the Open Science Framework (10.17605/OSF.IO/RFCTH). In order to reduce any error-related heteroscedasticity, HCC was log-transformed for further analysis^50^.

The lower limits of quantification of this assay method were below 0.1 pg cortisol per mg hair. The median coefficient of variation (CV) of all replicates was 4.9% [interquartile range: 1.3–13.9%].

### Statistical Analyses

All analyses were performed using JAGS 4.3.0^51^, R 3.4.2 statistical software^52^, and the R packages *psych* and *metafor*^53,54^. The internal consistencies of the three subjective stress measures were quantified as *ω* coefficients^55^. Retest stability *r*_*tt*_ was assessed based on autocorrelations of the weekly hassles and the perceived stress scales across the different assessment occasions. To increase the precision of these estimates, meta-analytical random effects modelling was used to pool the information.

The dynamic measurement model that was used to investigate all hypotheses is manifested by the following state Equation(1):

1 $${S}_{i,t}={S}_{i,t-1}+{\delta }_{i}+{\xi }_{i,t}$$

where S_i,t_ denotes the latent level of subjective stress in the i^th^ individual at the t^th^ assessment occasion, which depends on subjective stress at the previous assessment occasion S_i,t−1_. Moreover, δ_i_ represents the inter-individually varying, systematic change and ξ_i,t_ ~ Normal(0, σ_ξ_) represents the stochastic change of subjective stress across time. The corresponding observation Equation(2) is:

2 $${Q}_{i,j,t}={\lambda }_{j}{S}_{i,t}+{\xi }_{i,j,t}$$

where Q_i,j,t_ denotes the observed score of the j^th^ stress questionnaire (WHS_occur_, PSS, or TICS), and λ_j_ the respective factor loadings. Finally, ε_i,j,t_ ~ Normal(0, σ_ε_) represents measurement residuals, that is, all variance that was not related to subjective stress (incl. variance due to measurement error and confounding traits; see section 1.1). A detailed introduction to this so-called state-space (also known as dynamic linear) modelling approach is given in Chapter 9.4 of Jackman^56^, see also Auger-Méthé and colleagues^57^. The application of conceptually similar moment structure models has recently been advocated for developmental psychometrics because (1) they enable the unbiased estimation of the construct associations by separating true construct variance from construct-unrelated variance while (2) accounting for the potentially lagged impacts of constructs on their criteria^58,59^. All data and the commented R scripts of the reported analyses can be downloaded from the Open Science Framework (10.17605/OSF.IO/RFCTH). The model syntax is additionally provided as supplementary material.

Proceeding from the outlined model specification, the time series’ of self- and partner-reported subjective stress measures were jointly fitted by Monte-Carlo sampling from three Markov chains (20,000 iterations each). All stress measures were standardised before they were submitted to analysis. Gelman-Rubin diagnostics indicated the convergence of all chains $(\hat{R} < 1.01)$. The correlation between the stochastic change of self- and partner-reported latent subjective stress levels was directly inferred by putting a weakly informative inverse Wishart prior on the distribution of (σ_ξ_)^2^. By contrast, the correlation of the deterministic change of self- and partner-reported latent stress levels was inferred from the unconditional estimates of δ_i_. In conjunction with the factor loadings λ_i,j_, these correlations served to investigate if subjective stress is similarly reflected by the different questionnaires and informant perspectives. Finally, to investigate the temporal effect dynamics of subjective and objective stress measures, HCC was sequentially regressed on the time-varying estimates of self- and partner-reported stress levels S_t_. As HCC was hypothesized to be a retrospective indicator of objective stress, the regression coefficients of S_t_ were expected to be maximal after approx. half of the time period covered by the hair strands had elapsed (i.e., around the 6^th^ week of the 12 weeks sampling period).

## Results

### Properties of Subjective Stress Measures

The data of all assessed subjective stress measures and HCC are summarized in Table 2. For reasons of completeness, descriptive information on the secondary weekly hassle measures WHS_n_ and WHS_intens_ is also provided. Notably, most of the subjective stress measures covered approx. 65% of their possible score ranges. Only WHS_occur_ and WHS_intens_ measures covered a considerable smaller range portion of approx. 45%. On average, all participants (and their partners) reported ~12 hassles in the course of the preceding week.

**Table 2:** Descriptive information about the objective stress measure HCC and the subjective stress measures (points, pts) as reported by our participants and their partners.

No	Measure	Scaling	Informant	Min	Max	M	SD
1	HCC	pg/mg hair	Assay	0.48 (0)	18.24 (∞)	4.06	2.54
2	WHS_n,self_	pts/week	Self	2.58 (0)	21.50 (30)	11.83	3.96
3	WHS_n.partner_	pts/week	Partner	2.50 (0)	22.00 (30)	11.68	4.57
4	WHS_occur,self_	pts/week	Self	0.14 (0)	1.36 (4)	0.69	0.25
5	WHS_occur,partner_	pts/week	Partner	0.10 (0)	1.55 (4)	0.67	0.30
6	WHS_intens,self_	pts/week	Self	0.10 (0)	1.47 (4)	0.70	0.32
7	WHS_intens,partner_	pts/week	Partner	0.02 (0)	1.64 (4)	0.68	0.35
8	PSS_self_	pts/month	Self	1.33 (0)	32.67 (40)	13.91	5.20
9	PSS_partner_	pts/month	Partner	3.33 (0)	24.33 (40)	13.71	4.69
10	TICS_self_	pts/3 months	Self	1.00 (0)	39.00 (48)	13.72	7.46
11	TICS_partner_	pts/3 months	Partner	2.00 (0)	33.00 (48)	14.07	7.29

Weekly hassles (WHSn, WHSoccur, WHSintens) and Perceived Stress Scores (PSS) were averaged across all 12 weeks, hair cortisol (HCC) concentrations were averaged across all three 1 cm segments.

*Note*. Values in brackets indicate the possible range of the respective measure. HCC = hair cortisol concentration; WHS_n_ = number of reported weekly hassles; WHS_occur_ = mean score of weekly hassle occurrence; WHS_intens_ = mean score of weekly hassle intensity; PSS = sum score of the perceived stress scale; TICS = sum score of the TICS screening scale for chronic stress experience.

**Figure 3:**
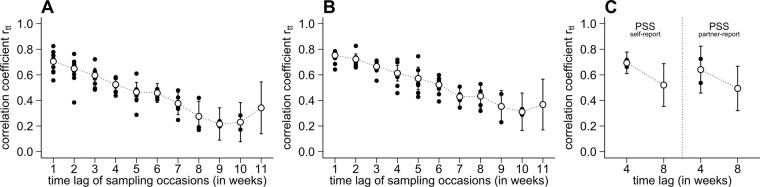
Autocorrelation plots of (**A**) self- vs. (**B**) partner-reported weekly hassle occurrence (WHS_occur_), and (**C**) perceived stress scores. Black dots indicate the point estimates of the Pearson correlation coefficients at each time lag, whereas the white dots are representing the meta-analytically pooled correlation coefficients (±standard error) for each time lag.

In the present study, self- and partner-reports showed no considerable differences with response to internal consistency and retest stability (see Table 3 for details). The TICS screening scale for chronic stress showed an internal consistency of *ω* = 0.91. Similar internal consistencies were found for the PSS (0.87 < *ω* < 0.89) and WHS_occur_ (0.80 < *ω* < 0.89). Thus, a considerable portion of variance in the WHS_occur_ may reflect a general inertia of environmental characteristics, and/or the individual disposition to report and perceive these characteristics as stressful^14^. With regard to retest stability, the WHS_occur_ ranged in between 0.17 < *r*_*tt*_ < 0.83. Figure 3A,B visualize these autocorrelations of the WHS_occur_ for both informant perspectives, that is, the change of retest stability with growing time lags between the assessment occasions. The PSS suggested retest stabilities of *r*_*tt*_ = 0.72 (one month) vs. 0.49 < *r*_*tt*_ < 0.52 (two months; see Fig. 3C).

**Table 3:** Pearson correlations coefficients for all subjective and objective stress measures reported by our participants or their partners.

	HCC	WHS_n,self_	WHS_n,partner_	WHS_occur,self_	WHS_occur,partner_	WHS_intens,self_	WHS_intens,partner_	PSS_self_	PSS_partner_	TICS_self_	TICS_partner_
HCC	*0.95*										
WHS_n,self_	0.17	*0.84*									
WHS_n,partner_	0.02	**0.26**	*0.88*								
WHS_occur,self_	0.27	0.91	**0.34**	*0.84*							
WHS_occur,partner_	0.08	**0.25**	0.94	**0.39**	*0.88*						
WHS_intens,self_	0.22	0.86	**0.29**	0.93	**0.36**	*0.85*					
WHS_intens,partner_	−0.01	**0.24**	0.89	**0.38**	0.95	**0.37**	*0.87*				
PSS_self_	0.11	0.38	**0.28**	0.52	**0.40**	0.52	**0.45**	*0.89*			
PSS_partner_	0.22	**0.16**	0.48	**0.34**	0.56	**0.31**	0.65	**0.54**	*0.87*		
TICS_self_	0.16	0.33	**0.27**	0.49	**0.34**	0.49	**0.39**	0.72	**0.44**	*0.91*	
TICS_partner_	0.04	**0.16**	0.42	**0.28**	0.53	**0.26**	0.60	**0.42**	0.70	**0.43**	*0.91*

*Bold font highlights the between-informant correlations. The matrix diagonal (italic font)*

lists the (mean) estimated internal consistency coefficients ω.

*Note*. HCC = hair cortisol concentration; WHS_n_ = number of reported weekly hassles; WHS_occur_ = mean score of weekly hassle occurrence; WHS_intens_ = mean score of weekly hassle intensity; PSS = sum score of the perceived stress scale; TICS = sum score of the TICS screening scale for chronic stress experience.

### Common Variance In Subjective Stress Measures

Table 3 lists the correlations between all averaged stress measures. With regard to the subjective stress measures, the largest cross-measure correlations were observed for the TICS and the PSS (0.70 < *r* < 0.72). By contrast, WHS_occur_ was only fairly associated with the TICS and the PSS (0.49 < *r* < 0.56), suggesting that the TICS and the PSS share additional variance due to factors/traits that are not reflected by the WHS_occur_.

In agreement with this notion, dynamic factor analysis revealed that latent subjective stress levels were best indicated by WHS_occur_. After full standardization, the factor loadings of self-reported stress amounted to λ_WHS,self_ = 0.87, λ_PSS,self_ = 0.49 and λ_TICS,self_ = 0.44, respectively. Similarly, the factor loadings of partner-reported stress levels amounted to λ_WHS,partner_ = 0.91, λ_PSS,partner_ = 0.53 and λ_TICS,partner_ = 0.47, respectively. Accordingly, 72%–81% of the variance in the PSS and the TICS scores were probably attributable to residual, stress-unrelated factors. Adjustment for participants’ sex reduced this portion of residual variance to 70%–74% in the PSS and the TICS scores. By contrast, the residual variance in WHS_occur_ only amounted to 17% to 21%, which is only slightly lower than the portion of error variance implied by its internal consistency estimates (see *section 3.1*). Adjustment for participants’ sex hardly reduced this residual variance in WHS_occur_ any further.

### Correspondence Of Self- And Partner Reports

According to Table 3, the self- and partner-reports of subjective stress were fairly associated, although the WHS_occur_ showed a lower association (r = 0.39) as compared to the PSS (*r* = 0.54) and the TICS (*r* = 0.43). The suggestively lower cross-informant association of the WHS was probably promoted by 20% to 45% additional variance in the partner-based reports (as indicated by their overall higher SDs reported in Table 2).

**Figure 4:**
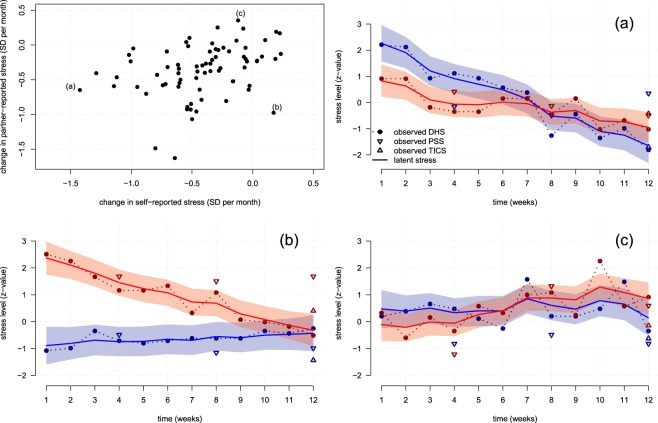
Systematic and stochastic changes of subjective stress measures (WHS, PSS, TICS) across time. The upper left panel shows the association of the systematic changes reported by both informants, with three exemplarily highlighted participants (**a**–**c**). The observed (dashed) and latent (solid) stress trajectories of these participants (blue: self-report, red: partner report) are shown in the remaining panels: (**a**) partner- and self-reported levels decrease, (**b**) partner-reported stress levels decrease, but self-reported stress levels increase, and (**c**) partner-reported levels increase but self-reported stress levels remain stable over time. The shaded areas indicate the 95% credible regions of the predicted (i.e., latent) subjective stress trajectories. WHS = reported frequency of weekly hassle occurrence; PSS = score of the perceived stress scale; TICS = score of the TICS screening scale for chronic stress experience.

Based on these data, we investigated to what extent the different measures of subjective stress (WHS_occur_, PSS, TICS) actually reflected the same covariance as a function of informant perspective. Regarding the systematic changes in latent stress levels across time, self- and partner-reports correlated weakly with *r*_δ_ = 0.34 (CI_95%_: 0.12–0.52, see Fig. 4). By contrast, the stochastic (transient) changes displayed a large correlation of *r*_ξ_ = 0.70 (CI_95%_: 0.55–0.84; see the exemplary couples shown in Fig. 4). These findings suggest that transient fluctuations in subjective stress levels are indeed captured by both, self- and partner-based reports. However, the majority of variance in latent subjective stress is attributable to inert (systematic) changes in subjective stress levels that seem to depend to a larger extent on the individual characteristics of the respective informant. On average and in both informants, the latent subjective stress levels tended to decrease across time and therefore, across the different assessment occasions (δ_self_ = −0.10 ± 0.09, δ_partner_ = −0.09 ± 0.09).

### Association Of Subjective Stress And Hair Cortisol

Table 3 also lists the correlations between all averaged subjective stress measures and the objective stress measure HCC. Notably, no subjective stress measure correlated significantly with HCC except for the self-reported WHS_occur_ (*r* = 0.27).

**Figure 5:**
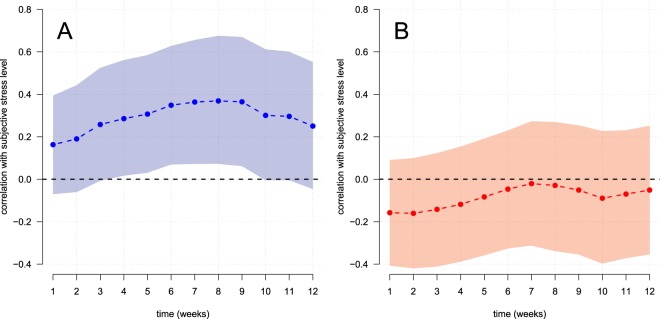
Trajectory of associations of hair cortisol levels (HCC in 3 cm of hair) and subjective stress levels that were inferred from (**A**) self- or (**B**) partner-reports of WHS_occur_, PSS, and TICS scores at the respective assessment occasion.

However, state-space modelling confirmed our expectation that the correlation between subjective stress and HCC depended on the time lag between the assessment occasions. The effect dynamics between the latent self- or partner-reported subjective stress levels and the manifest objective stress measure HCC are illustrated in Fig. 5A,B, respectively. As can be seen, subjective stress levels had the highest correlation with objective HCC if they were (1) based on self-report and (2) assessed between 3 to 8 weeks before hair sampling (max. α_self_ = 0.45, CI_95%_ = 0.10–0.80, corresponding to a max. *r* = 0.37, CI_95%_ = 0.07–0.68). By contrast, subjective stress levels inferred from the partner reports did not significantly predict objective HCC (max. α_partner_ = −0.02, CI_95%_ = −0.35–0.30, corresponding to a max. *r* = −0.02, CI_95%_ = −0.31–0.27). Adjustment for participant’s sex did not change these associations between subjective stress levels and HCC.

## Discussion

The present study was designed to investigate how self- and partner-reported subjective stress measures are related to objective HCC. When subjective measures were averaged across time, only the self-reported occurrence of weekly hassles (WHS_occur_) was considerably associated with HCC, whereas the PSS or the TICS score were not^29,38^. However, when all subjective stress measures were submitted to a dynamic factor analysis, it became evident that each of these subjective stress measures explained an incremental variance portion of the underlying, time-continuous processes (e.g., subjective appraisal). Nonetheless, the PSS and the TICS were less informative of subjective stress levels than the WHS_occur_, which highlights their above-mentioned, increased susceptibility to method bias (e.g., recall bias, and confounding)^12^. Interestingly, these findings generalized to both informant perspectives.

Importantly, only self-reported stress significantly predicted HCC (in 3 cm hair strands) with a maximal association found at a 4-week delay between the assessment of both stress measures (cf. Fig. 5). The attenuation of this association at the beginning and the end of the assessment period is consistent with a presumed, temporal delay between cortisol secretion in response to (subjective) *stress* and its reflection in HCC^28,29^. Therefore, the present data strongly support the retrospective character of cortisol measurement in hair and its (temporal) relation to subjective stress. Apart from this general support, however, the majority of HCC variance (i.e., 84%) seems to be attributable to other causes than subjective stress levels such as individual dispositions to synthesize cortisol. This interpretation agrees with recent evidence from twin studies, suggesting that 63% to 79% of HCC variance is attributable to additive genetic influences that were unrelated to subjective stress levels^15,60^.

From a methodological point of view, it is of particular importance that only the statistical modelling of the repeatedly assessed data unveiled the theoretically hypothesized effect dynamics between subjective stress levels and HCC. By contrast, the time-integrated measures neither provided such fine-graded information nor suggested a prominent correlation between subjective stress measures and HCC^28,38^. Because most previous studies also relied on simple correlations, objective stress measures (like HCC) were often found to barely reflect the same processes as self-report measures of subjective stress (like the PSS or the TICS)^1,28^. To the best of our knowledge, the present study is the first that empirically confirmed this central assumption – the *common cause hypothesis* – by showing that HCC (covering cortisol secretion across 12 weeks) is only predicted by subjective stress with a time lag of ~ 4 weeks (cf. *section 1.3*.). Thus, we provide novel evidence for the validity of HCC as a (retrospective) stress indicator^28,29^. Practitioners and inclined researchers should also consider this temporal perspective and thus optimize their assessment protocols by selecting the most suitable (i.e., longitudinal) designs and methods of data analysis.

As a by-product, the compiled data also provide tentative evidence against the so-called *stress-intensity hypothesis*^13,29^. Adapted to hair cortisol as a presumed objective measure of stress, the hypothesis predicts that only stressors of a certain intensity are capable of inducing (repeated) HPA-axis activity and accordingly, cortisol secretion and incorporation into scalp hair. In consequence, low-intensity stress is supposedly insufficient to increase HCC variance, which will necessarily reduce the probability of detecting an association between HCC and subjective stress measures^61^. However, in the present study we observed a dissociation between the PSS and the TICS compared to the subjective frequency of weekly hassle occurrence in predicting HCC. As this dissociation was probably attributable to the larger portion of stress-unrelated variance in the PSS and the TICS, the present findings are not necessarily in agreement with the *stress-intensity hypothesis* but highlight the methodological complications when retrospective self-report questionnaires covering comparably long time periods are used for stress assessment.

The statistical modelling also provided more detailed information that may help to explain the divergence of self- and partner-reported stress levels with regard to the prediction of HCC. While transient (stochastic) fluctuations of these stress levels where indeed captured by both informants (as indicated by their high correlation), the long-term (systematic) changes in self- and partner-reported subjective stress levels were only weakly associated. Accordingly, the lacking predictive value of partner-reported subjective stress may be attributable to individual dispositions of the participants or their partners that hardened the vicarious assessment of subjective stress. Such dispositions may lead to a differentially changing compliance of the participants themselves and their partners to repeatedly provide accurate data, which is a known issue in the context of repeated assessments in ambulatory settings^19,62^. In this regard, partner-reports may have been less motivating for maintaining the same reporting precision across a prolonged period of time. Although the identification of such moderators is a considerable challenge for future research on assessment methodology, our work suggests at least that individual differences in empathy unlikely contribute to the decreased validity of partner-reported subjective stress levels with respect to HCC (see supplementary data). However, we would also like to remark that these findings about cross-informant correlations are based on heterosexual couples and may therefore not generalize to other social dyads.

Finally, and irrespective of any individual disposition of the informant, the association between subjective and objective stress measures might also be confounded by environmental variables that jointly change HCC and subjective stress experiences. Notably, such variables may not necessarily attenuate but could as well inflate the association of subjective and objective stress measures. For example, it has been shown that an increasing exposure of hair to ultraviolet/sunlight radiation reduces HCC^63^. Increasing sunlight exposure, however, has also been demonstrated to reduce subjective stress experiences^64^. Conversely, physical activity increases cortisol turnover and sweating (and thereby the fraction of hair surface that is exposed to cortisol)^30,65^. At the same time, however, there is evidence for a stress-reducing effect of physical exercise^5,66–68^.

Proceeding from these examples, it becomes evident that the (presence or lack of) covariance in subjective and objective stress measures may actually arise from different causes. Accordingly, subjective and objective stress measures should ideally not be considered as fully interchangeable assessment tools. Instead multimethod stress assessment may provide distinct information about the mechanisms governing psychobiological health and pathology if the respective advantages and limitations of its components are appropriately taken into account. In this regard, the establishment of convergent validity using subjective and objective stress measures is to our mind the benchmark for robust inference on the presence of stress, whereas discriminant validity of subjective vs. objective stress measures should be considered with caution because it might as well be driven by stress-unrelated traits or method artifacts.

## Supplementary information

Appendix
