# Supplementary material for: The psychometric properties and temporal dynamics of subjective stress, retrospectively assessed by different informants and questionnaires, and hair cortisol concentrations
Source: Sci Rep. 2019 Jan 31;9:1098. doi: 10.1038/s41598-018-37526-2 (PMC6355861; doi:10.1038/s41598-018-37526-2)
Supplement: Supplementary file 1 — Appendix [file 41598_2018_37526_MOESM1_ESM.pdf]

The psychometric properties and temporal dynamics of subjective stress, retrospectively assessed by different informants and questionnaires, and hair cortisol concentrations

Lisa J. Weckesser[1], Friedericke Dietz[1], Kornelius Schmidt[1], Juliane Grass[1],
Clemens Kirschbaum[1], Robert Miller[1,2]

[1]Faculty of Psychology, Technische Universität Dresden, Dresden, Germany
[2]Unit Epidemiology, Statistics, and Exposure Modeling, Federal Institute for Risk Assessment, Berlin, Germany

APPENDIX A

Weekly Hassle Scale (WHS)

The presented WHS was comprised of 30-items that were selected from three different original daily hassle scales: *Liste Alltäglicher Streßereignisse (LAS,* 87 items),[44] the *Revised Daily Hassle Scale* (RDHS, 63 items / 7 subscales),[42] and the *Revised University Student Daily Hassle Scale* (RUSHS, 57 items / 11 subscales).[43] After completing the WHS for themselves (self-report instruction), all participants were asked to complete it vicariously for their partners (partner-report instruction). Notably, the WHS incorporates at least one item from each of the 18 original subscales of the exhaustive RDHS and RUSHS, but only requires ~10 minutes for completion. To translate suitable RDHS/RUSHS items into German, we selected the conceptually matching items from the German LAS scale. The translations of the remaining items into German were not literal but coextensive. The actually presented German items are reported in square brackets.

Instruction self-report: Below you will find a list of possible everyday events. Please indicate first of all whether an event occurred last week and then how much it affected you. Please do not skip any event.

Instruction partner-report: Now, please think about your partner. Please indicate first how often your partner has experienced each particular event during the last week and second, how stressful it was for him/her. If you are not sure about any event, please choose the option that you think is most appropriate.

**Nr**	**Hassle**	**Original Scale(s)**
1	Too many things to do<br>[Zu viel zu tun]	RUSHS/RDHS
2	Exhaustion<br>[Erschöpfung oder Energiemangel]	RUSHS/RDHS
3	Relaxation problems<br>[Entspannungsprobleme]	RUSHS/RDHS/LAS
4	Lack of sleep<br>[Zu wenig Schlaf]	RUSHS
5	Not enough free time<br>[Zu wenig Freizeit]	LAS
6	Not enough time for family<br>[Zu wenig Zeit für die Familie]	RUSHS/LAS
7	Too many social obligations<br>[Zu viele soziale Verpflichtungen]	RUSHS/RDHS
8	Loneliness<br>[Einsamkeit]	RUSHS/RDHS
9	High demands of others<br>[Erwartungen und Ansprüche der Kinder, Partner, Familie oder Freunde]	RUSHS/RDHS
10	High demands of oneself<br>[Eigene Erwartungen und Ansprüche]	RUSHS
11	Concerns about future<br>[Sorgen über die Zukunft]	RDHS/LAS
12	Concerns about family<br>[Sorgen um die Kinder, Partner, Familie oder Freunde]	RDHS
13	Concerns about the own physical appearance<br>[Gedanken über das äußere Erscheinungsbild]	RUSHS/RDHS/LAS
14	Concerns about the meaning of life	RDHS/LAS

	[Gedanken über den Sinn des Lebens]	
15	Job dissatisfaction/ not having a job	RDHS/LAS
	[Unzufriedenheit mit der Arbeit oder damit, keine Arbeit zu haben]	
16	Compatibility of family and work	RUSHS/RDHS
	[Problem, Arbeit und Familie zu vereinbaren]	
17	Conflicts at work/ university/ school	RDHS/LAS
	[Konflikt am Arbeitsplatz, im Studium, in Ausbildung oder Schule]	
18	Time pressure at work/university/school	RUSHS/RDHS
	[Termindruck]	
19	Decision problems	LAS
	[Schwierigkeiten, Entscheidungen zu treffen]	
20	Health problems	RDHS/LAS
	[Körperliche Beschwerden]	
21	Financial problems	RUSHS/RDHS
	[Schulden oder finanzielle Probleme]	
22	High living expenses	LAS
	[Hohe Lebenshaltungskosten]	
23	Sexual problems	LAS
	[Sexuelle Probleme]	
24	Social conflicts	RUSHS/LAS
	[Konflikt mit den Kindern, Partner, Familie oder Freunden]	
25	Noise disturbance	RDHS
	[Lärmbelästigung]	
26	Troubles about traffic/parking	RUSHS/RDHS/LAS
	[Ärger über den Verkehr oder Parkplatzsuche]	
27	Discrimination due to gender	RUSHS/RDHS
	[Diskriminierung aufgrund des Geschlechts]	
28	Discrimination due to religion	RUSHS/RDHS
	[Diskriminierung aufgrund von religiöser oder ethischer Überzeugung]	
29	Discrimination due to race/ethnicity	RUSHS/RDHS
	[Diskriminierung aufgrund der Herkunft]	
30	Discrimination due to age	RUSHS
	[Diskriminierung aufgrund des Alters]	
